# Expanded Group Additivity Framework for Thermochemical Prediction of Fluorocarbons and PFAS from Large-Scale DFT Data

**DOI:** 10.1021/acs.jpca.6c03256

**Published:** 2026-08-18

**Authors:** Samuel Eccles, Steven Pellizzeri

**Affiliations:** Department of Chemistry and Biochemistry, 2344 Eastern Illinois University, 600 Lincoln Avenue, Charleston, Illinois 61920, United States

## Abstract

Fluorinated molecules, including per- and polyfluoroalkyl substances (PFAS), present persistent challenges for thermochemical characterization due to limited experimental data, strong carbon–fluorine bonding, and the rapidly expanding size and diversity of fluorinated chemical space. While density functional theory (DFT) calculations can provide useful thermochemical data for individual fluorinated species, their routine application becomes increasingly impractical as molecular size, conformational complexity, and the number of distinct PFAS compounds continue to grow. Existing Benson-type group additivity schemes provide limited resolution for fluorinated environments, restricting their applicability to modern fluorinated and PFAS-relevant systems. Here, we develop a chemically resolved group additivity (GA) framework for fluorinated and PFAS-relevant species by fragmenting DFT-derived thermochemistry for 3070 molecules. This approach expands the available fluorinated Benson-type group library from 14 to 159 local environments and integrates the resulting groups within the Python Group Additivity (pGrAdd) framework. 10-fold cross-validated regression against DFT data yields root-mean-square deviations (RMSDs) of 8.14 kcal·mol^–1^ for enthalpy and 10.05 cal·mol^–1^·K^–1^ for entropy, which are reduced to 2.55 kcal·mol^–1^ and 6.36 cal·mol^–1^·K^–1^, respectively, following application of independently defined nongroup interaction correction terms in pGrAdd. Comparison with available experimental thermochemical data shows improved agreement and reduced bias compared to legacy Benson group libraries. This expanded fluorinated GA framework enables scalable and chemically interpretable thermochemical predictions for fluorinated and PFAS-relevant species, supporting kinetic modeling and mechanistic studies where direct electronic structure calculations are feasible but not scalable.

## Graphical abstract

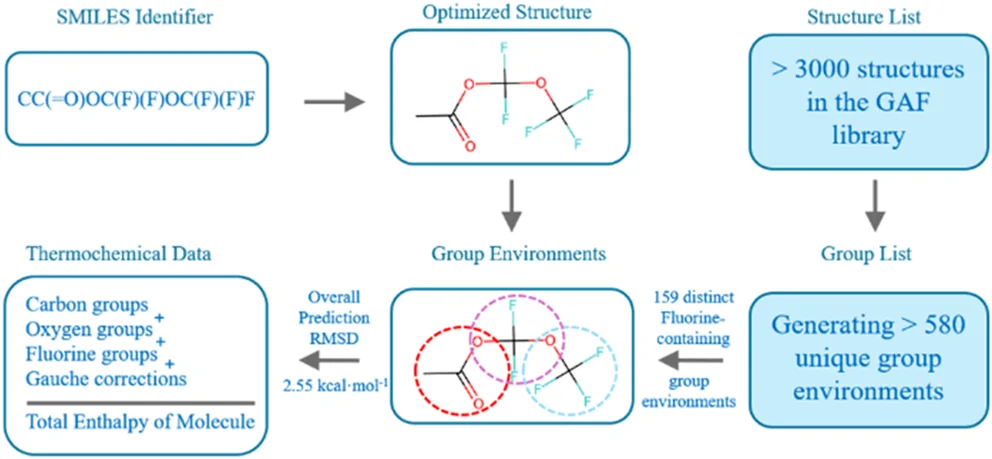

## 1 Introduction

Fluorocarbons and per- and polyfluoroalkyl substances (PFASs) constitute a chemically resilient class of compounds with extensive industrial use, long environmental lifetimes, and increasing regulatory scrutiny.^1–6^ Their exceptional thermal stability, hydrophobicity, and resistance to degradation result in environmental persistence and widespread distribution, resulting in measurable human exposure and documented associations with adverse health outcomes.^7–10^

Experimental characterization of fluorocarbon thermochemistry is hindered by the high bond dissociation energies of carbon–fluorine linkages and the unique electronic structure of heavily fluorinated environments.^11–14^ As a result, calorimetric and equilibrium measurements are scarce, particularly for ionized and polyfunctional PFAS, and are restricted to small, neutral molecules.^15^ In contrast, PFAS encompass a wide range of chain lengths, substitution patterns, functional headgroups, and charge states, far exceeding the limited set of species for which reliable, high-quality thermochemical data are currently available. This mismatch between chemical diversity and available data necessitates scalable, transferable thermochemical estimation methods capable of extending reliable prediction across fluorinated systems. The usefulness of this information extends beyond thermochemistry and is relevant for predicting environmental and climate-relevant metrics for the assessment of known and next-generation fluorochemicals.^16–20^

Density functional theory (DFT) calculations can, in principle, provide useful enthalpies, entropies, and heat capacities for fluorinated species and have been successfully applied to small fluorocarbons and selected PFAS molecules.^11,12,21–24^ However, the routine application of DFT across the PFAS chemical space is increasingly constrained by molecular size, conformational flexibility, and the rapidly growing number of distinct PFAS structures. Using the definition of a PFAS molecule by Gaines et al.,^25^ the number of known PFAS molecules now encompasses tens of thousands of structures.^26^ These practical limitations are compounded by known functional- and basis set sensitivities in highly fluorinated environment systems.^27–35^ As a result, scalable thermochemical estimation methods remain essential for extending reliable predictions across fluorinated systems beyond the reach of direct electronic structure calculations applied on a case-by-case basis.

Recent developments have sought to use data-driven machine- and deep-learning approaches to make predictions of thermodynamic properties across chemical space.^36–38^ For example, Chen et al. introduced a deep-learning-based increment theory, in which graph neural networks learn atom-centered thermochemical contributions while preserving the additive structure characteristic of traditional increment methods.^39^ While such approaches benefit from large, diverse training data sets, the available fluorocarbon thermochemical data remain comparatively sparse, particularly for larger and more structurally complex PFAS species. Because of limits in available data, a regression-based group additivity framework was instead adopted in this work using a purely geometric grammar-based approach through classical Benson group additivity methods.

Group additivity (GA) offers a computationally efficient framework for estimating thermochemical properties by decomposing molecules into transferable local environments, whose contributions can be reused across structurally diverse species.^40,41^ This framework and its modern implementations have been extensively applied to hydrocarbons, oxygenates, and nitrogen-containing species, enabling rapid generation of thermodynamic parameters for kinetic modeling and mechanism development.^40–44^ However, the applicability of existing GA schemes to fluorinated systems remains limited. Most available Benson-type libraries contain only a small number of fluorine-containing groups, often restricted to simple substitution patterns.^11,12,22^ As a result, these schemes lack the chemical resolution required to describe strongly inductive perfluoroalkyl environments, fluorinated ether linkages, and charged PFAS headgroups.

To address these limitations, we developed an expanded fluorinated group library and integrated it with the Python Group Additivity (pGrAdd) framework developed by Vlachos and co-workers.^43^ pGrAdd provides a structured and internally consistent implementation of Benson-type group additivity augmented by nongroup interaction corrections that account for nonlocal effects such as ring strain, steric interactions, and intermolecular electrostatics. Importantly, these corrections are defined independently of the present data set and have been used previously across diverse nonfluorinated chemical classes, reducing the risk of data set-specific bias. Here, we use DFT-derived thermochemistry for more than 3,000 fluorocarbon and PFAS-relevant molecules to generate a chemically resolved library of fluorine-containing Benson-type groups and correction terms. By embedding the newly developed fluorinated library within this established framework, the present approach keeps the interpretability of classical group additivity while systematically improving the performance for chemically complex and sparsely sampled fluorinated environments.

## 2 Computational Methods

All quantum mechanical density functional theory (DFT) electronic structure calculations were done in Gaussian16 (Revision C.01),^45^ employing the MN15 global hybrid meta-GGA functional,^46^ the def2-TZVP basis set,^47^ and ultrafine integration grids for all numerical integrations. The MN15 functional was selected for its balanced performance in thermochemistry and its reported accuracy for standard enthalpies of formation, which is important for determining group-additivity parameters. In its original benchmark study, MN15 yielded the lowest mean unsigned error among 82 density functionals for a database of 313 single-reference energetic data (1.85 kcal mol^–1^) while also maintaining excellent performance for noncovalent interactions (0.25 kcal mol^–1^), supporting its use as a broadly transferable method for the chemically diverse fluorinated structures examined in this work.^46,48^ No empirical dispersion corrections were added, as the MN15 functional has been shown to perform comparably with dispersion-corrected functionals for systems without significant noncovalent interactions.^46^ The def2-TZVP basis set provides reliable performance for main-group elements, including highly electronegative atoms such as fluorine.^47^

Geometry relaxations were performed using the Berny algorithm using the GEDIIS optimization method with the RMS force convergence criterion set to 10^–5^ Hartree Bohr^–1^. Geometry optimizations were performed for all atoms. Prior to geometry relaxations, initial three-dimensional molecular coordinates were generated for the corresponding SMILES^49–51^ string using RDKit’s ETKDG algorithm^52^ followed by a short MMFF/UFF relaxation. This provides reasonable starting configurations for geometry optimization using DFT but does not constitute a full conformational sampling workflow. As a result, the data set may contain a mixture of global and higher-energy local minima.

Vibrational frequency analyses were performed on all optimized structures to confirm the absence of any imaginary frequencies and to obtain zero-point and thermal corrections. Harmonic vibrational frequencies were scaled by a uniform factor of 0.975 to correct systematic functional- and basis-set-dependent errors inherent to the harmonic approximation.^31^ Although this scaling factor was originally derived for MN15 with a closely related triple-ζ basis set, scaling factor databases indicate that MN15 vibrational frequencies exhibit only modest sensitivity to basis set choice, and use of the closest available literature value is not expected to introduce significant systematic error. Any residual systematic error associated with this approximation is expected to be effectively absorbed into the regressed group-additivity parameters. Low-frequency vibrational modes (<100 cm^–1^), which can lead to artificial inflation of vibrational entropies, were treated using a quasi-harmonic correction in which all frequencies below this threshold were raised to 100 cm^–1^.^30,53,54^

## 3 Results and Discussion

### 3.1 Overview of the Fluorinated pGrAdd Workflow

The complete workflow for constructing and integrating fluorinated group additivity parameters into the pGrAdd framework is summarized in Figure 1. DFT calculations were performed for a library of 3070 fluorocarbon- and PFAS-related structures, followed by statistical-mechanical conversion of the electronic energies and vibrational spectra into ideal-gas thermodynamic functions using the Python Multiscale Thermochemistry Toolbox (pMuTT).^44^

**Figure 1:**
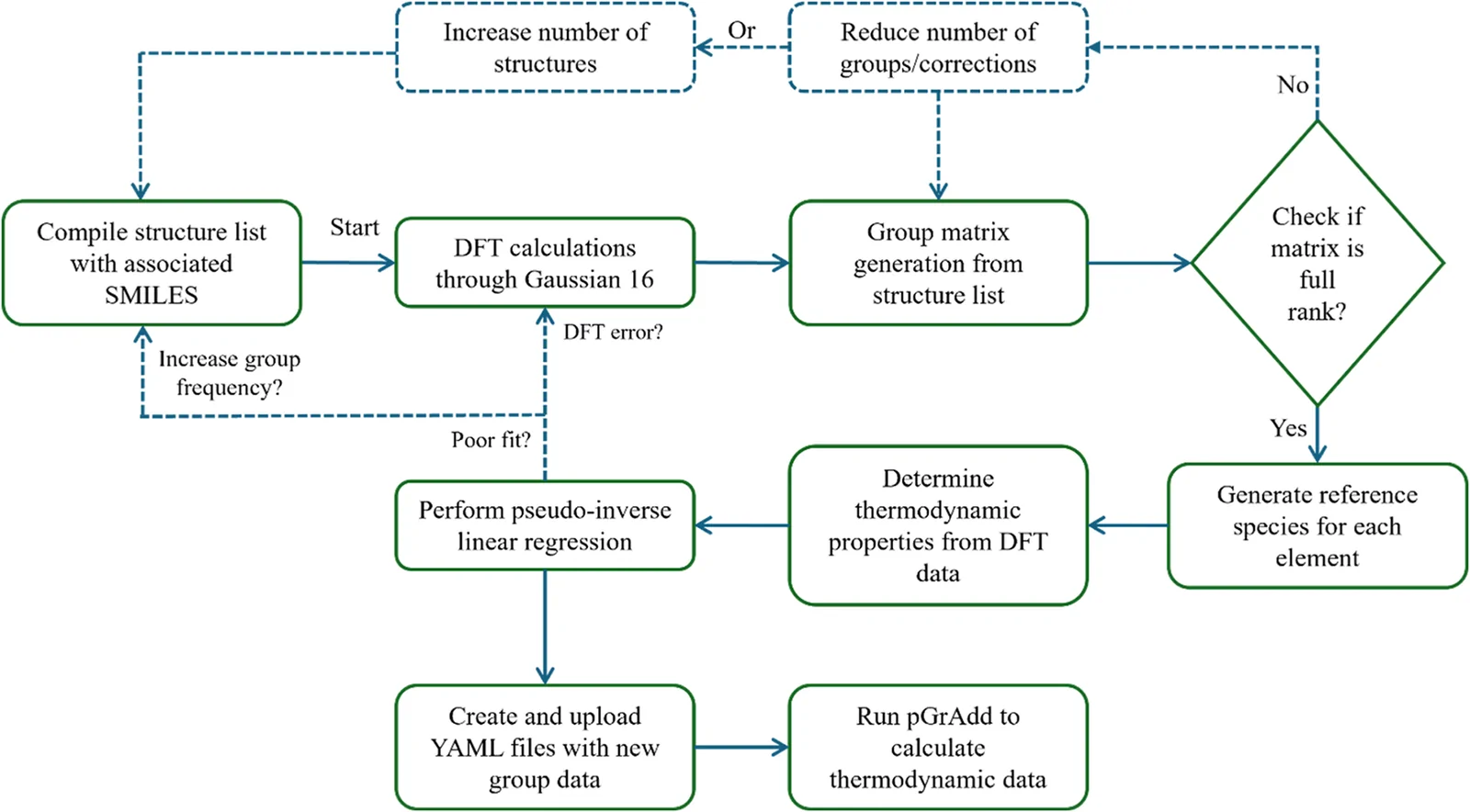
Schematic representation of the workflow for development and integration of the fluorinated group-additivity (GAF) library within the pGrAdd framework. DFT for 3070 fluorocarbon and PFAS-relevant species was converted to ideal-gas thermodynamic functions and aligned to an experimental reference scale via element-specific reference corrections. Molecular structures were fragmented using an extended attribute-grammar scheme to generate fluorinated Benson-type group contributions, which were incorporated into pGrAdd to enable automated thermochemical prediction with optional nongroup interaction correction terms.

These values were aligned to an experimental enthalpy scale via element-specific reference corrections and later used to parametrize an expanded fluorinated group-additivity (GAF) library via pseudoinverse linear regression.

### 3.2 Generation and Expansion of Fluorinated Groups

Group additivity parameters for fluorinated and PFAS-relevant species were derived from DFT thermochemistry and implemented with the pGrAdd framework.^43^ The overall procedure consists of a representative molecular data set, grammar-based group definition and fragmentation of fluorinated local environments, generation of thermochemical training data, and regression of group contributions.

The molecular data set was primarily drawn from the U.S. EPA DSSTox PFAS inventory (version 5) encompassing over 10,000 PFAS structures and augmented with an additional 297 fluorinated species designed to introduce recurring functional motifs and underrepresented chemical environments.^55^ All molecules were encoded using SMILES^49–51^ strings to ensure consistent mapping between the DFT thermochemical data and grammar-based group decomposition. A complete list of all structures used in the regression is provided in the Supporting Information.

Group definitions and molecular fragmentation were implemented using the Silver extensible attribute-grammar system^56^ based on the pGrAdd formalism used by Vlachos and co-workers.^43^ The grammar was extended to explicitly represent fluorinated carbon centers, multiple halogen substitution patterns, charged moieties, and PFAS-relevant functional groups. This extended scheme was implemented as a pGrAdd scheme file, enabling the automated and chemically consistent fragmentation of fluorinated species. Representative examples of the extensible attribute-grammar syntax are shown in Figures S1 and S2.

Thermochemical target data were generated from DFT electronic energies and vibrational frequencies via statistical-mechanical analysis using pMuTT.^44^ Ideal-gas enthalpy, entropy, and constant-pressure heat capacity were evaluated over 300–1500 K, consistent with the classical Benson group-additivity framework.^41^ Element-specific reference corrections were applied to align DFT-derived enthalpies with experimental reference scales using evaluated gas-phase data.^57,58^ Details of thermochemical target construction, reference species selection and corrections, group definitions, and fragmentation rules are provided in Sections 2 and 3 as well as Figure S3, Tables S1, and S2 in the Supporting Information.

Application of the extended attribute-grammar scheme^56^ resulted in a substantial expansion of available fluorine-containing Benson-type groups. The original Benson framework includes only 14 fluorinated groups, largely restricted to simple substitution patterns. In contrast, the present work generates 159 distinct fluorine-containing local environments. The number of groups added to different atom-centered group families, and examples of these groups, are provided in Table 1. This represents one of the largest internally consistent fluorinated GA libraries derived from a single DFT data set.

**Table 1:** Classification of the 159 Fluorinated Group Additivity Fragments Developed In This Work, Organized by Central Atom or Structural Motif^a^

group family	count	examples
fluorine-centered	8	F(C), F(N), F(C[*d*])
carbon-centered	106	C(C)(F)3, C(Cl)(F)(H)(O), C[*t*](C[*t*])(F)
oxygen-centered	4	O(C)(F), O(F)(P), O(F)(S)
nitrogen-centered	9	N(C)(F)2, N(F)2(S), N(F)(H)(O)
sulfur-centered	7	S(C)(F), S(F)(N), S(F)(O)(O[*d*])
phosphorus-centered	4	P(C)(F)(H), P(C)3(F)2, P(F)(O[*d*])
aromatic-containing	10	F(C[A]), C(C[A])(F)3, C(C)(C[A])(F)(O)
charge-containing	11	F(C[-]), C(F)3(N[+]), C(C)(C[-])(F)2

a The table lists the number of fragments within each family and representative examples illustrating the chemical environments captured by the regressed group additivity framework.

The expanded library reflects increased chemical resolution rather than purely numerical growth. For example, while conventional schemes treat −CF_3_ as a distinct group, the present framework decomposes this motif into a carbon-centered environment C-(C)­(F)_3_ and three explicit F-(C) interactions. This fine-grained representation preserves overall energetic contributions while explicitly capturing substitution-dependent inductive effects, bond polarization, and local electronic structure.

Group occurrence across the data set is inherently uneven, with common environments appearing thousands of times, while specialized groups having charged or highly substituted fluorinated centers may only occur once or twice. This variability has important implications for regression uncertainty and predictive accuracy, addressed through cross-validation and nongroup interaction correction terms, as discussed in Section 3.3.

### 3.3 Regression of Group Contributions and Cross-Validation

Group contributions for enthalpy, entropy, and heat capacity were obtained by linear least-squares regression of the group-occurrence matrix against DFT-derived thermochemical target, with predictive performance evaluated using 10-fold k-fold cross-validation.^59,60^ Full details of the regression formulation, cross-validation procedure, and outlier determination are provided in the Supporting Information (Section S4). Enthalpy and entropy were calculated at 300 K to maintain consistency with the original Benson group additivity work, and heat capacity was evaluated over a 300–1500 K range for the same reason.

Data set-wide root-mean-square deviation (RMSD) values obtained from cross-validated regression without nongroup interaction corrections are summarized in Table S3, with corresponding parity plots for enthalpy and entropy predictions shown in Figure 2a,b, respectively. Predicted enthalpies of formation show strong linear correlation with DFT reference values across the full data set, with an overall RMSD of 8.14 kcal·mol^–1^ (Table S3). The absence of systematic curvature or offset indicates that the regressed local group contributions capture the dominant energetic trends associated with fluorinated substitution patterns. The magnitude of these errors is consistent with expectations for a local additivity model trained on quantum-chemical thermochemistry.^33,61^

**Figure 2:**
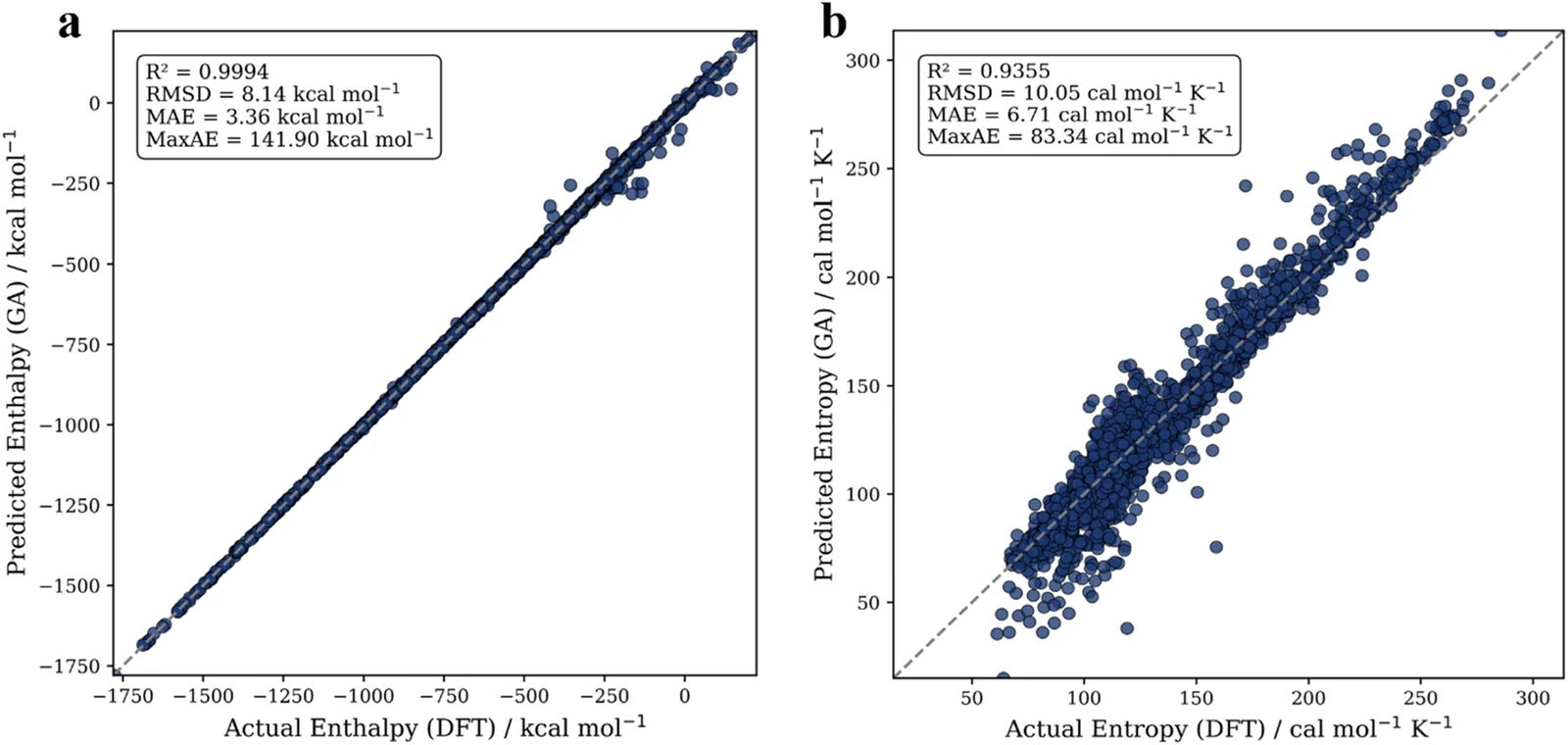
Parity plots for cross-validated thermochemical predictions obtained from the fluorinated group-additivity regression without nongroup interaction corrections. (a) Predicted versus DFT-derived enthalpies of formation (kcal·mol^–1^) and (b) predicted versus DFT-derived entropies (cal·mol^–1^·K^–1^) using 10-fold cross-validation. The dashed line indicates ideal 1:1 agreement. Strong linear correlation is observed for both properties, with increased scatter in entropy reflecting sensitivity to low-frequency vibrational modes and conformational effects not captured by purely local group contributions.

Entropy prediction exhibits a higher RMSD of 10.05 cal·mol^–1^·K^–1^ (Figure 2b). This behavior reflects the sensitivity of absolute entropies to low-frequency vibrational and torsional modes, as well as the intrinsic limitations of a purely local group-additivity representation for flexible fluorinated chains.^27^ Despite this increased scatter, the entropy parity plot maintains strong linearity and exhibits no pronounced systematic bias, indicating that the extracted group contributions reproduce the dominant local entropy trends across chemically diverse fluorinated species.

For a data set comprising several thousand fluorocarbons and PFAS-relevant molecules with wide variation in chain length, functionalization, and conformational flexibility, RMSD on the order of 8 kcal·mol^–1^ for enthalpy and 10 cal·mol^–1^·K^–1^ entropy are consistent with reported performance of modern group-additivity thermochemistry models.^62,63^ The largest deviations were noticed to be associated with molecules dominated by sparsely sampled or chemically atypical local environments, where limited statistical support increases parameter uncertainty, as shown in Section S5 and Table S4 of the Supporting Information, contributing to the high maximum absolute error.

The regressed fluorinated group parameters were subsequently integrated into pGrAdd (version 2.9.12) as a YAML-formatted group library, enabling applications of the framework’s nongroup correction terms from Benson and colleagues.^40,41,43,64^ These corrections account for nonlocal effects originating from next-nearest neighbor interactions that cannot be included in local groups. Briefly, these were determined through the direct comparison of experimental data of similar systems with and without the desired correction. From this comparison, corrections such as ring strain can be determined and added into the estimation of the thermodynamic properties of the molecular compounds.

Incorporation of nongroup corrections (NGC) leads to a substantial improvement in predictive accuracy, as illustrated by the parity plots in Figure 3. Following application of these corrections and excluding a single statistical outlier identified via Grubbs’ test,^65^ the enthalpy RMSD is reduced from 8.14 to 2.55 kcal·mol^–1^ (Figure 3a, *R* ^2^ = 0.99). Compared to the uncorrected regression (Figure 2a), the corrected enthalpy parity plot exhibits markedly reduced scatter and improved symmetry about the 1:1 line, indicating that systematic deviations associated with nonlocal interactions have been substantially reduced.

**Figure 3:**
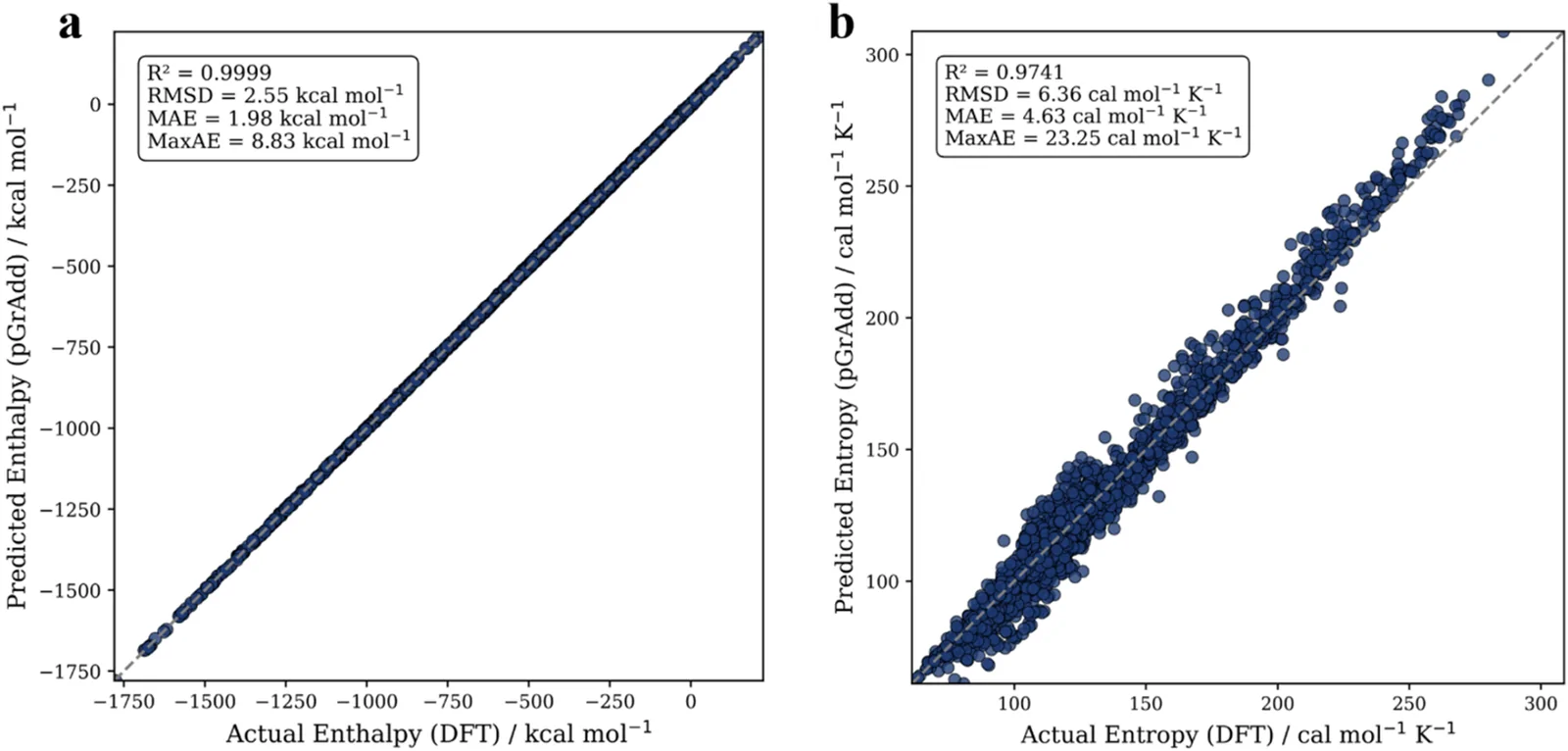
Parity plots for thermochemical predictions obtained after application of pGrAdd nongroup interaction corrections. (a) Predicted versus DFT-derived enthalpies of formation (kcal·mol^–1^) and (b) predicted versus DFT-derived entropies (cal·mol^–1^·K^–1^) for the fluorinated data set. Nongroup interaction corrections accounting for steric, electrostatic, and conformational effects not recoverable from local group additivity alone. A single statistical outlier identified via Grubbs’ test was excluded. Inclusion of nongroup interaction corrections substantially reduces scatter and systematic deviations relative to the uncorrected predictions shown in Figure 2.

Entropy prediction shows a similar improvement upon application of nongroup interaction corrections. As shown in Figure 3b, the entropy RMSD decreases from 10.05 to 6.36 cal·mol^–1^·K^–1^, accompanied by a noticeable reduction in scattering relative to the raw group-additivity predictions (Figure 2b) as well as significant improvements in MAE and MaxAE. Unlike enthalpy, entropy depends not only on local functional groups but also on molecular flexibility, conformational accessibility, and vibrational modes, leading to a higher RMSD seen in both Figures 2 and 4. The improved linearity and reduced bias indicate that the correction framework captures a significant portion of conformational, electrostatic, and collective vibrational effects that are compressed or neglected in a strictly local additive representation. Importantly, these improvements are defined independently of the present fluorinated data set, reducing the risk of data set-specific overfitting.

**Figure 4:**
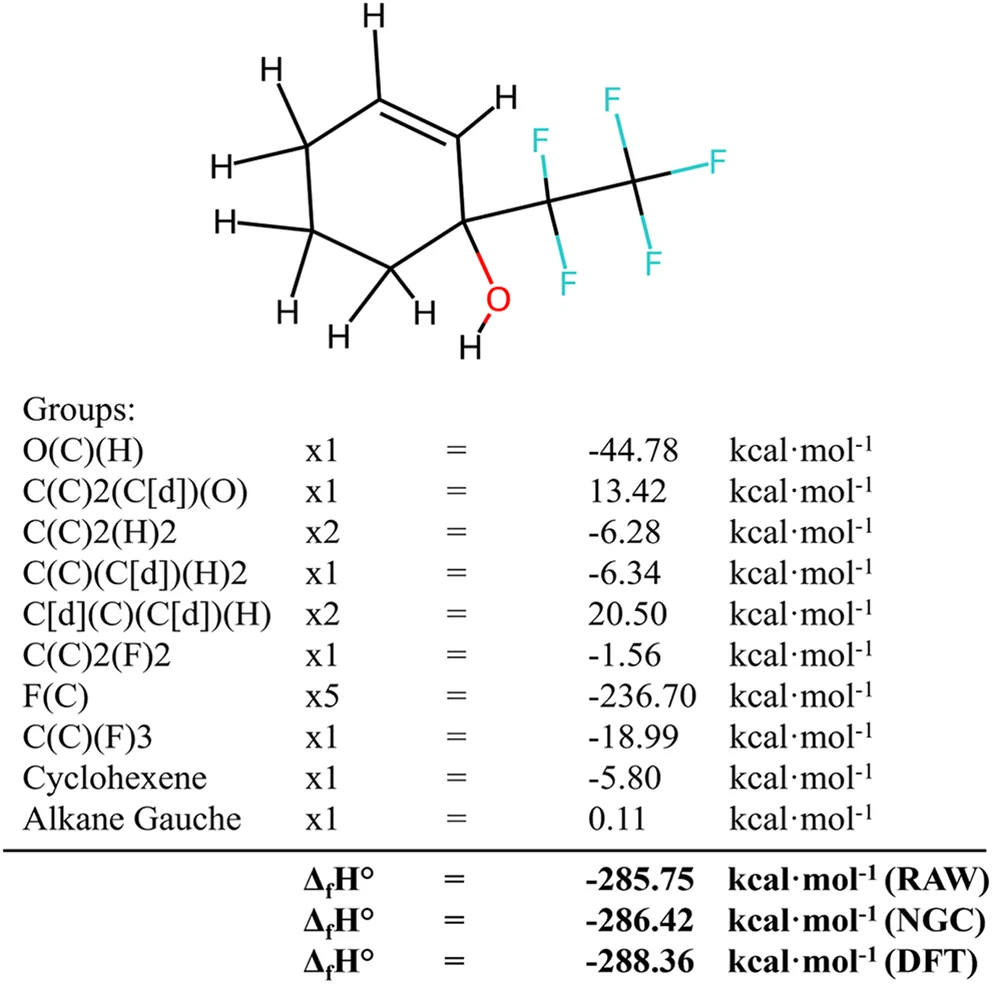
Example group-additivity decomposition and enthalpy reconstruction for a representative fluorinated PFAS alkenol using the expanded fluorinated pGrAdd (GAF) framework. The molecular structure is fragmented into chemically resolved local Benson-type groups using the extended attribute-grammar scheme. Each group contributes independently to the raw (“RAW”) group-additivity enthalpy, which is augmented by Benson nongroup corrections (“NGC”). Comparison with the DFT-derived enthalpy illustrates how nonlocal corrections systematically reduce residual error for chemically complex fluorinated species.

### 3.4 Physical Interpretation and Illustrative Case Studies

The following case studies illustrate how chemically resolved local groups and NGC terms act together within the expanded fluorinated pGrAdd framework. These examples are intended to provide physical interpretation of model behavior rather than statistical validation, which is addressed separately in Section 3.5.

For molecules dominated by well-sampled and chemically typical local environments, raw group additivity predictions already provide accurate thermochemical estimates. In such cases, application of nongroup corrections provides only a minor change, showing that local contributions capture the dominant energetic effects. In contrast, chemically complex species having sparsely sampled, charged, or highly substituted fluorinated environments exhibit noticeably larger deviations when using local groups alone (Table S6), reflecting the influence of nonlocal inductive, electrostatic, and conformational interactions.

An example illustrating the effects of the addition of NGC corrections is provided in Table 2 for two representative fluorinated PFAS species. *1,1,2,2,3-Pentafluorobutane* (ID# 290), which is composed of well-represented local environments, exhibits group-additivity (GA) enthalpy prediction error below 1 kcal·mol^–1^ relative to DFT using pure local group regression only. In contrast, *amino­(difluoroimino)­methanaminium* (ID# 2948), which contains more unique and sparsely sampled local environments, shows a raw group-additivity error of 15.12 kcal·mol^–1^ that is reduced to 3.75 kcal·mol^–1^ following application of nongroup interaction corrections. These examples highlight the role of NGCs in accounting for electronic contributions that are not recoverable from purely local group additivity. This is particularly true for fluorinated and charged systems, where strong inductive effects, hyperconjugation, and intramolecular interactions play a significant role.^66–68^ Importantly, nongroup interaction corrections do not overcorrect molecules dominated by well-sampled environments, but instead selectively improve predictions for chemically challenging cases.

**Table 2:**
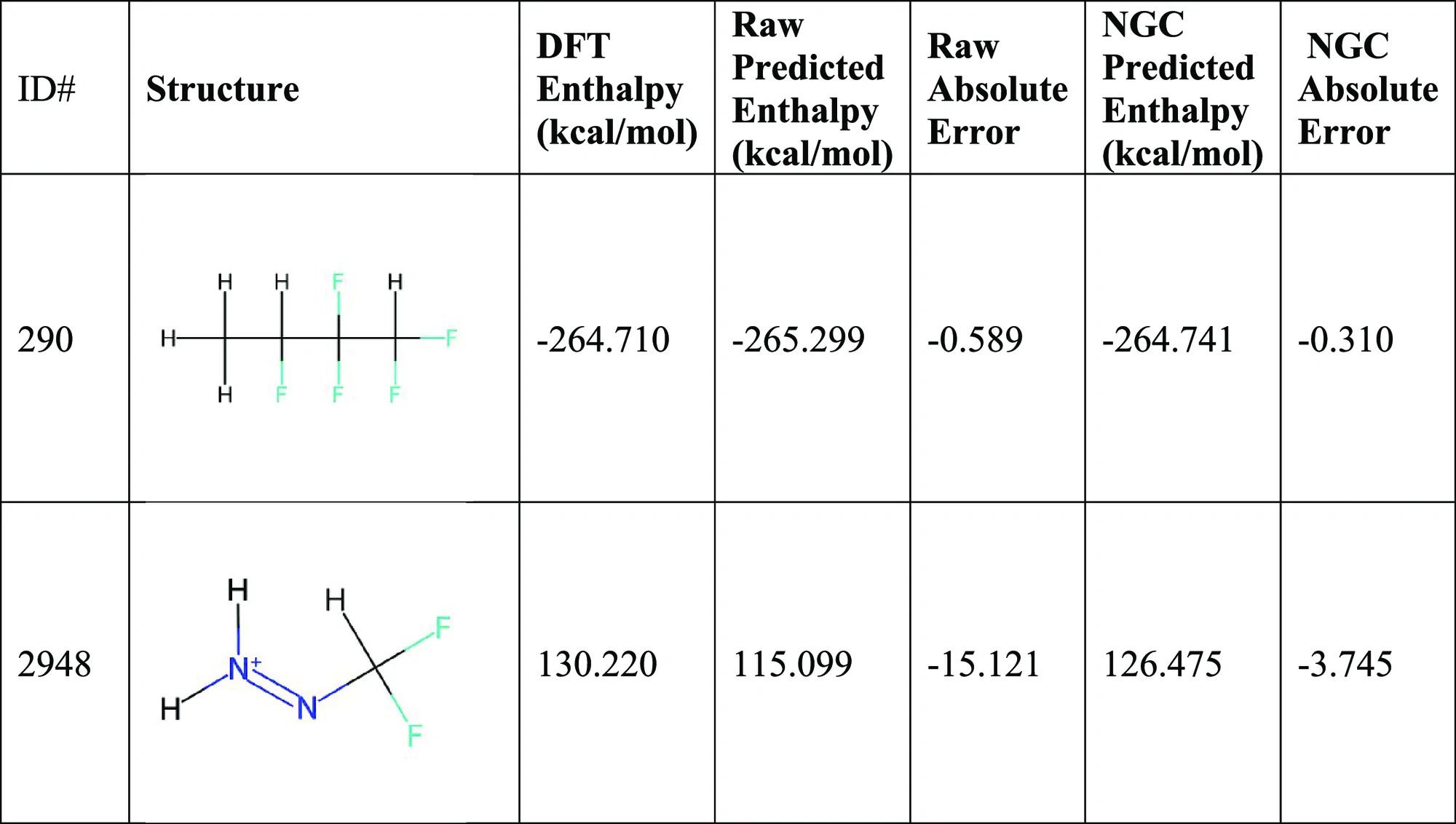
Comparison of Raw Group-Additivity (GA) and Nongroup Correction (NGC) Predictions for Representative Fluorinated PFAS Species^a^

a Raw predictions include only local Benson-type group contributions, while NGC predictions incorporate non-nearest-neighbor correction terms. Absolute errors are reported relative to DFT-derived enthalpies of formation. Application of nongroup interaction corrections substantially reduces errors for chemically complex and sparsely sampled environments, while only minor improvements are observed for molecules dominated by well-represented local groups.

Further insight into this behavior is provided by the explicit group-additivity decomposition shown in Figure 4 for the fluorinated PFAS alkenol 1-(1,1,2,2,2-pentafluoroethyl)­cyclohex-2-en-1-ol (ID# 2511). Fragmentation using the extended attribute-grammar scheme yields chemically resolved local environments, including explicit fluorinated carbon centers and C–F interaction groups. Summation of these local contributions captures substitution-dependent energetic trends but leaves residual deviations associated with non-nearest-neighbor interactions. Application of the pGrAdd correction framework systematically accounts for these residual effects, shifting the predicted enthalpy toward the DFT reference value.

Together, these case studies demonstrate the improved predictive performance of the pGrAdd GAF library compared with the pure regression model for the fluorinated compounds evaluated in this work. The combination of chemically resolved local group definitions and independently parametrized nongroup interaction corrections provides a more complete description of molecular environments than regression alone while preserving the chemically interpretable nature of the group additivity framework.

### 3.5 Validation against Experimental Thermochemistry

Model performance was assessed through comparison with available experimental gas-phase enthalpies of formation for fluorocarbon and PFAS-relevant molecules reported in the NIST Chemistry WebBook.^69^ All structures used for validation correspond to species built using groups previously examined in the Benson literature, enabling a direct comparison between the legacy Benson group-additivity scheme^40^ and the present fluorinated pGrAdd model trained specifically on fluorinated chemical space.

Because the experimental data set is limited and biased toward small, lightly fluorinated molecules, validation is evaluated in terms of error magnitude and systematic bias rather than pointwise agreement. The distribution of enthalpy prediction errors relative to experimental values is shown in Figure 5 for both the expanded fluorinated pGrAdd framework and the Benson group-additivity scheme. Across this validation set, the expanded fluorinated pGrAdd model yields an RMSD of 3.52 kcal·mol^–1^ relative to experiment, representing an approximately 50% reduction in error compared to the Benson scheme (RMSD = 6.87 kcal·mol^–1^).

**Figure 5:**
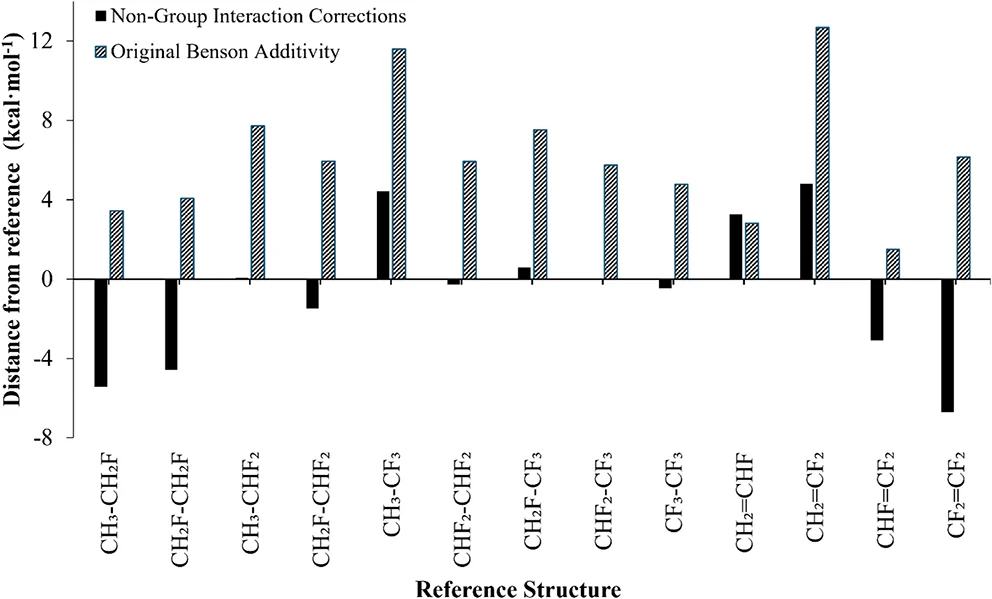
Distribution of enthalpy prediction errors relative to experimental gas-phase enthalpies of formation from the NIST Chemistry WebBook for the legacy Benson group-additivity scheme and the expanded fluorinated pGrAdd framework. Errors are defined as predicted minus experimental values. The Benson scheme exhibits broad error dispersion and a pronounced positive bias, whereas the pGrAdd predictions show a narrower, more symmetric error distribution centered near zero, indicating reduced systematic bias and improved consistency for fluorinated species.

In addition to lower overall error, the pGrAdd predictions show a narrower and more symmetric error distribution centered near zero. In contrast, the Benson scheme shows a pronounced positive bias and highly variable error distribution with deviations exceeding ±10 kcal·mol^–1^ in this limited data set. The reduced spread and near-zero centering of the pGrAdd error distribution reflects improved predictive consistency for fluorinated molecules.

Despite the small size and compositional bias of the experimental data set (Table S7), preventing definitive assessment across the full PFAS chemical space, the observed reduction in both error magnitude and systematic bias demonstrates improved predictive performance for the fluorinated compounds evaluated in this study. Additional comparison with experimental and high-level composite calculations employing conformer sampling, B3LYP/def2-TZVP-D3­(BJ) vibrational analyses, DF-MP2/aug-cc-pVQZ geometries, and local CCSD­(T)/aug-cc-pVQZ single-point energies is provided in the Supporting Information (section 6, Table S8, and Figure S4), where similar trends are observed, further supporting the robustness of the expanded fluorinated group-additivity framework. Comparison to DLPNO–CCSD­(T)/CBS values for varying functional groups ranging from C_2_–C_8_ in chain length (Table S9) was also done to assess accuracy as chain length increases.

## 4 Conclusion

We have developed an expanded group-additivity framework for fluorinated and PFAS-relevant species by combining a large, internally consistent MN15/def2-TZVP DFT thermochemical data set with an extended attribute-grammar formalism implemented within the pGrAdd framework. The resulting fluorinated group library substantially increases chemical resolution relative to legacy Benson-type schemes, expanding the number of fluorine-containing local environments from 14 to 159 while retaining the transparency and interpretability of classical group additivity.

Cross-validated regression against DFT-derived thermochemistry shows that the expanded fluorinated group library captures the dominant local energetic and entropic trends across diverse fluorinated chemical space. Incorporation of independently defined Benson nongroup correction terms substantially reduces residual errors associated with nonlocal steric, electrostatic, and conformational effects, yielding improved predictive accuracy without introducing data set-specific fitting bias.

Although validation with more structurally diverse PFAS systems will become feasible as additional experimental thermochemical data become available, the observed improvements in predictive accuracy and reduced systematic bias demonstrate the effectiveness of the expanded fluorinated group library for the fluorinated compounds evaluated in this study.

Overall, these results demonstrate that chemically resolved local group definitions, combined with independently parametrized nonlocal corrections, provide a scalable and interpretable framework for thermochemical estimation across fluorinated and PFAS-relevant chemical space. This expanded framework enables rapid generation of thermochemical data for applications such as kinetic modeling and mechanistic analysis, where direct electronic structure calculations become increasingly impractical as the molecular size, conformational complexity, and chemical diversity continue to expand.

## Supplementary Material



## Notes

Detailed description of the expanded fluorinated group-additivity framework, including reference species selection and reference corrections, regression methodology and cross-validation statistics, RMSD analyses, validation against NIST and high-level theoretical data, scheme file examples for fluorinated and charged environments, outlier analysis, supplementary tables and figures, and references. A complete list of molecular structures, SMILES strings, and links to all DFT data and analysis scripts are hosted on the PellizzeriLab GitHub repository (https://github.com/PellizzeriLab/Computational-Methods-for-Rapid-Fluorocarbon-Characterization) and are released under the MIT License. Users are requested to cite this manuscript when using these materials (PDF)
