# Supplementary material for: Expanded Group Additivity Framework for Thermochemical Prediction of Fluorocarbons and PFAS from Large-Scale DFT Data
Source: J Phys Chem A. 2026 Aug 18;130(34):6825–34. doi: 10.1021/acs.jpca.6c03256 (PMC13528296; doi:10.1021/acs.jpca.6c03256)
Supplement: Supplementary file 1 [file jp6c03256_si_001.pdf]

Supplementary Materials for

Expanded Group Additivity Framework for Thermochemical Prediction of Fluorocarbons and PFAS from Large-Scale DFT Data

Samuel Eccles[a] and Steven Pellizzeri[*,a]

[a] Department of Chemistry and Biochemistry, Eastern Illinois University, 600 Lincoln Avenue, Charleston, IL 61920, USA

*corresponding author email: spellizzeri@eiu.edu

Scope of the Supporting Information..S4

S1. DFT Data and Python Code Availability..S4

S2. Thermochemical Target Construction S4

S3. Group Definition and Fragmentation Rules S8

S4. Regression and Cross-Validation Methodology..S9

S5. Structure Error Analysis S11

S6. Validation against Experimental and High-Level Quantum Chemical Data (B3LYP/def2-TZVP-D3(BJ) vibrational analyses, DF-MP2/aug-cc-pVQZ geometries, and local CCSD(T)/aug-cc-pVQZ single-point energies) S11

S7. Supplementary Tables S13

Table S1. Reference species selected to anchor DFT-derived thermochemistry to experimentally evaluated gas-phase enthalpy scales for each element present in the dataset. Species were chosen based on the availability of high-quality experimental thermochemical data, closed-shell electronic structure, and relevance to fluorinated chemical environments. Species containing multiple elements contribute simultaneously to the reference correction for each constituent element. S14

Table S2. Element-specific reference corrections obtained by aligning DFT-based enthalpies of formation at 298 K to experimentally evaluated thermochemical data using the pMuTT framework. Reported values (in Hartree) represent cumulative molecular reference offsets applied to ensure internal consistency across the dataset and should not be interpreted as isolated atomic energies. S14

Table S3. Dataset-wide root-mean-square deviation (RMSD) values for enthalpy, entropy, and temperature-dependent heat capacities obtained from 10-fold cross-validated group-additivity regression using the expanded fluorinated pGrAdd framework. Reported RMSDs quantify aggregate predictive performance across the full fluorocarbon and PFAS dataset prior to application of long-range corrections and do not reflect errors for individual molecules. S15

Table S4. Highest maximum absolute error structures (MaxAE) (enthalpy) for pseudo-inverse linear regression of fluorinated structure list. MaxAE was used to check errors in structures and groups to ensure accuracy in group fragmentation and prediction.. ......S20

Table S5. Highest maximum absolute error structures (MaxAE) (enthalpy) for pGrAdd GAF library predictions of fluorinated structure list. MaxAE was used to check errors in structures and groups to ensure accuracy in group fragmentation and prediction.........S20

Table S6. Comparison of like-structures containing varying degrees of group occurrence attributing to the absolute error in prediction accuracy of the regression model.

Comparison structure FC1(F)C(=O)NC(=O)C1(F)F was chosen as it was already present in the dataset and differed only by one atom to FC1(F)C(=O)N(Br)C(=O)C1(F)F.. ....S20

Table S7. Experimental gas-phase enthalpies of formation at 298 K for fluorinated hydrocarbons obtained from the NIST Chemistry WebBook and associated literature sources. These species were used for comparison with predictions from the expanded fluorinated pGrAdd framework and to provide direct benchmark comparison to classical Benson-type group additivity schemes. S20

Table S8. Comparison of predicted enthalpies of formation from the expanded fluorinated pGrAdd group-additivity frameworkwith experimental and high level composite quantum chemical results (B3LYP/def2-TZVP-D3(BJ) vibrational analyses, DF-MP2/aug-cc-pVQZ geometries, and local CCSD(T)/aug-cc-pVQZ single-point energies) reported by Paulechka et. al.[11] Agreement with both experiment and high-level computation provides and independent benchmark and indicates good transferability within the small-chain fluorinated domain S21

Table S9. Comparison of predicted enthalpies of formation from the expanded fluorinated pGrAdd group-additivity framework with high level composite qauntum chemical results (DLPNO-CCSD(T)/CBS) reported in literature by Melin et.al.[12] Data provides a benchmark for the pGrAdd GAF library against a range of functional groups (alkane, alcohol and alkanoic acid) as well as chain length (C2-C8). S22

S8. Supplementary Figures S23

Figure S1. Example of Silver language rulesets used to identify fragments for an iodine center illustrating the definition of halogenated local environments within the fluorinated group-additivity framework. Groups are defined using an extensible attribute-grammar formalism that systematically captures fluorinated substitution patterns while preventing overlap or misrepresentation of chemically distinct environments. S23

Figure S2. Example of Silver language rulesets used to identify fragments for cationic nitrogen environments relevant to PFAS chemistry. The attribute-grammar formalism enables explicit differentiation of bonding patterns, coordination states, and charge environments required for chemically consistent fragmentation of ionized species. S23

Figure S3. Grubbs' test analysis applied to cross-validated residuals following application of pGrAdd long-range corrections. The test was used as a diagnostic tool to identify statistically anomalous predictions rather than as a definitive exclusion criterion, acknowledging assumptions of approximate normality and single-outlier behavior. ...S24

Figure S4.Residual comparison between predicted gas-phase enthalpies of formation and experimental reference values reported by Paulechka et al.[12] Residuals (Predicted − Experimental) are shown for both the expanded fluorinated pGrAdd framework and the original Benson group-additivity scheme. The dashed horizontal line denotes perfect agreement with experiment. Across this experimental dataset, the pGrAdd model exhibits reduced systematic deviation relative to the Benson scheme, yielding an RMSD of 5.58 kcal·mol^{-1} compared to 8.33 kcal·mol^{-1} for the legacy groups. These results demonstrate that the expanded fluorinated group library provides more accurate and consistent thermochemical predictions for fluorinated species.. ....S25

References S26

Scope of the Supporting Information

This Supporting Information provides detailed documentation of the data, reference anchoring procedures, regression methodology, and validation analyses underlying the expanded fluorinated group-additivity framework described in the main text. Supplementary tables and figures support the construction, parameterization, and evaluation of the fluorinated pGrAdd library, while full datasets and analysis scripts are made publicly available to ensure reproducibility.

S1. DFT Data and Python Code Availability

A full structure list with associated SMILES codes along with the data used in the regression analysis as well as all Python scripts used for group generation and regression analysis are contained within the PellizzeriLab GitHub page linked here:

PellizzeriLab/Computational-Methods-for-Rapid-Fluorocarbon-Characterization: Supporting information for the manuscript - Computational Methods for Rapid Fluorocarbon Characterization (https://github.com/PellizzeriLab/Computational-Methods-for-Rapid-Fluorocarbon-Characterization).

S2. Thermochemical Target Construction

Gas-phase thermochemical target data used for group-additivity regression were derived from DFT electronic energies and vibrational frequencies using statistical-mechanical analysis implemented in the Python Multiscale Thermochemistry Toolbox (pMuTT)[1] Harmonic vibrational frequencies were scaled using the uniform scaling factor described in Section 2.1 of the main text prior to thermochemical evaluation. pMuTT subsequently uses zero-point energy corrected values when computing thermochemical data in addition to the frequency scaling factor.

pMuTT specifically converts the DFT electronic energies and vibrational frequencies from Gaussian 16 into temperature-dependent thermodynamic functions through statistical mechanics using molecular partition functions. For an ideal gas molecule, the enthalpy at temperature (T) is calculated as

$$H(T) = E_{elec} + ZPE \ + H_{trans}(T) + H_{rot}(T) + H_{vib}(T) + RT$$

where (E_{elec}) is the DFT electronic energy, (ZPE) is the zero-point energy, and the remaining terms are the translational, rotational, and vibrational thermal contributions obtained from statistical thermodynamics. The entropy is similarly obtained as

$$S(T) = S_{trans}(T) + S_{rot}(T) + S_{vib}(T) + S_{elec}$$

where the electronic contribution to entropy is defined as

$$S_{elec} = R(\ln \omega_0 + 0)$$

ω_0 is the spin multiplicity and the Gibbs free energy is calculated from

$$G(T) = H(T) - TS(T)$$

using the standard rigid-rotor harmonic-oscillator approximation implemented in pMuTT.

The translational, rotational, and vibrational contributions to both enthalpy and entropy can be obtained using the appropriate molecular partition functions and thermodynamic functions. To obtain the enthalpy of translations ($H_{trans}(T)$) the following form is used

$$H_{trans}(T) = U_{trans}(T) + RT$$

where the internal energy of the translational motion $\left(U_{trans}(T)\right)$ is defined as

$$U_{trans}(T) = \frac{3}{2}RT$$

The entropy of translation $\left(S_{trans}(T)\right)$ is found using the translational partition function (q_t) using the mass of the molecule (m) and a defined pressure (P)

$$q_t = \left(\frac{2\pi m k_B T}{h^2}\right)^{\frac{3}{2}} \frac{k_B T}{P}$$

$$S_{trans}(T) = R\left(\ln q_t + 1 + \frac{3}{2}\right)$$

Like translation, molecular partition functions can be used to find rotational contributions to both the enthalpy(H_{rot}) and entropy(S_{rot}).

$$H_{rot}(T) = U_{rot}(T) = \begin{cases} 0 \; if \; monoatomic \\ RT \; if \; linear \\ \frac{3}{2} RT \; if \; non-linear \end{cases}$$

$$S_{rot}(T) = \begin{cases} 0 \; if \; monoatomic \\ \log\left(\frac{T}{\sigma} \prod_i \frac{1}{\Theta_{\mathrm{R,i}}}\right) + 1 \; if \; linear \\ \log\left(\frac{\sqrt{\pi}}{\sigma} \prod_i \left(\frac{T^3}{\Theta_{\mathrm{R,i}}}\right)^{\frac{1}{2}}\right) + \frac{3}{2} \; if \; non-linear \end{cases}$$

Where σ is the molecular symmetry number and Θ_R is the characteristic rotational temperature defined as

$$\Theta_R = \frac{h^2}{8\pi^2 I k_B}$$

Where I is the moment of inertia.

Finally, the vibrational contribution is defined using the molecular partition function as

$$q_V = \prod_K \frac{1}{1 - e^{-\frac{\Theta_{v,K}}{T}}}$$

Where $\Theta_{\mathrm{v,K}}$ is the characteristic vibrational temperature defined for each vibrational mode, K.

$$\Theta_{v,K} = \frac{hv_K}{k_B}$$

The vibrational contribution to the enthalpy (H_{vib}) and entropy (S_{vib}) is then found as:

$$H_{vib} = R\sum_{K} \Theta_{v,K} \left(\frac{1}{2} + \frac{1}{e^{\frac{\Theta_{v,K}}{T}} - 1}\right)$$

$$S_{vib} = R\sum_{K} \frac{\frac{\Theta_{v,K}}{T}}{e^{\frac{\Theta_{v,K}}{T}} - 1} - \ln\left(1 - e^{-\frac{\theta_{v,K}}{T}-1}\right)$$

To align the DFT-derived thermochemistry with an experimental enthalpy scale, a set of reference species with evaluated gas-phase thermochemical data (e.g., NASA polynomials[2] or Shomate parameters[3]) was selected from the NIST Chemistry WebBook,[4] with at least one reference species chosen for each element present in the dataset. Reference species were selected based on the availability of high-quality experimental thermochemical data, closed-shell electronic structure, and chemical relevance to fluorinated environments. Species having multiple elements contribute simultaneously to the reference correction for each constituent element and should not be interpreted as isolated atomic references. The complete list of reference species and corresponding correction values is provided in **Tables S1 and S2**.

Using these reference species, pMuTT was employed to generate temperature-dependent thermochemical properties by combining DFT electronic energies with statistical-mechanical treatments of vibrational, rotational, and translational degrees of freedom. DFT-derived enthalpies at 298 K were aligned to evaluated experimental values using additive reference offsets, ensuring internal thermochemical consistency across the dataset. The resulting reference energies represent cumulative molecular offsets and should not be interpreted as isolated atomic quantities.

Following application of reference corrections, gas-phase thermochemical properties were evaluated over the temperature range 300–1500 K and assembled into target vectors corresponding to enthalpy (H), entropy (S), and constant-pressure heat capacity (C_p). These thermochemical target vectors serve as the dependent variables used in the group-additivity regression described in **Section S3**.

Outlier detection was performed as a diagnostic step using Grubbs' test at the 95% confidence level,[5] applied to cross-validated residuals following application of long-range corrections within the pGrAdd framework. This procedure was used solely to identify statistically anomalous predictions and not as a definitive exclusion criterion. Details of the outlier analysis are provided in **Figure S3**.

S3. Group Definition and Fragmentation Rules

Group-additivity parameters in the expanded fluorinated pGrAdd[6] framework are defined using a grammar-based molecular fragmentation scheme through the Silver extensible attribute-grammar system.[7] In this framework, each group corresponds to a chemically distinct local environment centered on a specific atom and defined by its immediate bonding topology, elemental identity, substitution pattern, and charge state.

Groups are constructed using a central-atom formalism, in which the atom of interest serves as the group center and its bonded neighbors and bond orders are enumerated to capture chemically meaningful local structure while avoiding unnecessary over-parameterization. This approach ensures that groups represent transferable local bonding motifs rather than global molecular structure, preserving applicability across chemically diverse fluorinated and PFAS-relevant species.

Chemical uniqueness and mutual exclusivity of groups are enforced through the attribute-grammar formalism, which guarantees that each atom in a molecule is assigned to a

single, well-defined group and prevents overlapping or redundant group assignments during fragmentation. Grammar rules distinguish environments based on coordination number, bond order, substituent identity, and formal charge, ensuring consistent treatment of neutral and ionized species. Fluorinated carbon centers, heteroatoms, multiply substituted environments, and charged moieties are treated explicitly to capture strong inductive effects, bond polarization, and charge stabilization characteristic of fluorinated chemistry.

Wildcard symbols ($) are used selectively within group definitions to denote chemically equivalent substituent positions where explicit differentiation does not improve predictive accuracy. This controlled use of wildcards limits proliferation of sparsely sampled groups while maintaining sufficient chemical resolution. Variable atomic connectivity, such as the ability of nitrogen to adopt multiple coordination states and bond orders are handled through distinct grammar rules to prevent overlap and misrepresentation of chemically distinct environments.

Application of this grammar-based fragmentation scheme yields a unique, non-overlapping set of local groups for each molecule, which collectively reconstruct molecular thermochemistry through linear additivity. Representative examples of group definitions and fragmentation rules for halogenated and charged environments are provided in **Figures S1 and S2**.

S4. Regression and Cross-Validation Methodology

Group-additivity parameters were obtained by linear least-squares regression of the grammar-defined local groups described in **Section S3** against the DFT-derived thermochemical target vectors described in **Section S2**. For each molecular species i, the thermodynamic property y_i (enthalpy of formation, entropy, or constant-pressure heat capacity at a given temperature) is expressed as a linear combination of group contributions,

$$\mathbf{G}a \approx y$$

where $\mathbf{G}$ is the group-occurrence matrix of dimension $N_s \times N_g$, N_s is the number of molecular species, N_g is the number of distinct Benson-type groups, a is the vector of group parameters, and y is the vector of molecular thermochemical values.

Because the number of molecular species exceeded the number of distinct groups $(N_s \gg N_g)$, the regression system was overdetermined. Group parameters were therefore obtained by computing the minimum-norm least-squares solution using the Moore–Penrose pseudoinverse to compute the minimum-norm least-squares solution.[8] Regression was performed independently for enthalpy, entropy, and constant-pressure heat capacity at each temperature considered.

Predictive performance was assessed using k-fold cross-validation (10-fold), applied at the molecular level.[9,10] The full dataset was randomly partitioned into ten approximately equal subsets. For each fold, group parameters were regressed using nine subsets (training set), and thermochemical predictions were generated for the remaining subset (test set). Dataset-wide predictive accuracy was quantified using root-mean-square deviations (RMSDs) computed over all held-out predictions. This approach provides an unbiased minimum-norm solution for the group contributions but does not intrinsically regularize sparsely sampled environments. Consequently, RMSD values reported in **Table S3** reflect aggregate model performance, while uncertainty for low-occurrence groups remains higher and should be interpreted cautiously. RMSD values in **Table S3** were extracted directly from analysis code found inside provided Jupyter notebooks.

Following incorporation of long-range correction terms within the pGrAdd framework, a single statistical outlier was identified using Grubbs' test at the 95% confidence level and excluded from the corrected parity plots presented in the main text; regression coefficients were

otherwise retained unchanged. This treatment ensures transparency in data handling while avoiding refitting or dataset-specific tuning of group parameters.

The regressed group-additivity parameters were subsequently written to a YAML-formatted group library and incorporated as an input file for the pGrAdd framework, enabling automated thermochemical prediction using the expanded fluorinated group set. Additional discussion of regression performance and the impact of long-range correction terms is provided in Section 3.3 of the main manuscript.

S5. Structure Error Analysis

In order to ensure the pGrAdd GAF library was accurately describing group environments and evaluate if the program was correctly decomposing molecules into all its components correctly, structure error analysis was performed. This takes the 5 highest error structures, looks at the individual group make-up, does this match the SMILES structure and what is the occurrence of the group. This led to a noticeable pattern that the high error structures are composed of low-occurrence groups, and the lower the number of datapoints for that group, especially for the simple regression model, the higher the error in the structure. The highest error structures and the associated groups and occurrences can be found in **Tables S4** and **S5**. A comparison between like structures with differing group occurrences can be found in **Table S6** for visual comprehension.

S6. Validation against Experimental and High-Level Quantum Chemical Data (B3LYP/def2-TZVP-D3(BJ) vibrational analyses, DF-MP2/aug-cc-pVQZ geometries, and local CCSD(T)/aug-cc-pVQZ single-point energies).

In addition to validation against experiment, the expanded pGrAdd framework was also evaluated against both experimental and high-level composite quantum chemical data to provide an independent benchmark of predictive accuracy. Specifically, predicted gas-phase

enthalpies of formation obtained using the expanded fluorinated group-additivity library were compared with experimental and composite quantum mechanical reference values reported by Paulechka and Kazakov. [11]

The critically evaluated enthalpies of formation reported by Paulechka were selected as an independent reference dataset because they combine available experimental thermochemical measurements with high-level *ab initio* calculations, providing rigorous benchmark values for small, fluorinated molecules. Predicted enthalpies from the expanded fluorinated pGrAdd framework were generated using the same group parameters derived from the full fluorocarbon and PFAS dataset, without any refitting to the Paulechka reference set.

The comparison between pGrAdd predictions and the Paulechka reference values is summarized in **Table S5** and illustrated in **Figure S4**. Across this subset of fluorinated species (C2-C3), the expanded fluorinated group-additivity framework shows reduced systematic bias and lower error magnitude relative to the legacy Benson group-additivity scheme. Agreement with experimental and calculated reference values support the transferability of the regressed fluorinated group parameters beyond the DFT training set and indicates consistent performance for chemically related fluorinated functional classes.

This comparison incorporates both experimental measurements and high-level computational data, providing a more robust assessment of model performance than was previously possible. The agreement observed across independent reference sources demonstrates that the updated framework captures the dominant energetic trends governing fluorinated thermochemistry more reliably than the legacy Benson scheme. To compare the ability of the pGrAdd GAF library to accurately predict varying functional groups and chain length data from Melin et. al was used as a benchmark.[12] Three homologous series were considered: perfluorocarboxylic acids, perfluoroalkanes, and fluorinated alcohols, spanning

chain lengths from two to eight carbon atoms. Across all three series, the pGrAdd predictions closely reproduced the DLPNO-CCSD(T)/CBS reference values for the shorter chain compounds, with errors generally below 2 kcal·mol^{-1}. As chain length increased, a gradual increase in absolute error was observed, reaching approximately 5–6 kcal·mol^{-1} for the longest members of each series. Importantly, this trend was consistent regardless of functional group, indicating that the model maintains a stable level of predictive performance across chemically distinct fluorinated families. The systematic nature of the error suggests a modest accumulation of group additivity contributions with increasing molecular size rather than a breakdown in transferability of the GAF parameters. Overall, these results demonstrate that the pGrAdd model remains robust across a broad range of fluorinated compounds, with only a gradual reduction in accuracy as molecular size increases.

S7. Supplementary Tables

Supplementary Tables S1–S9 provide reference species definitions, correction values, regression performance metrics, and validation datasets supporting the analyses described in Sections S2 and S3.

Table S1. Reference species selected to anchor DFT-derived thermochemistry to experimentally evaluated gas-phase enthalpy scales for each element present in the dataset. Species were chosen based on the availability of high-quality experimental thermochemical data, closed-shell electronic structure, and relevance to fluorinated chemical environments. Species containing multiple elements contribute simultaneously to the reference correction for each constituent element.

Element	Chosen Reference Species
**H**	H_2O
**C**	CH_2FCHF_2
**N**	NH_3
**O**	CH_3OCH_3, CH_3COOH
**F**	F_2, CF_4, C_2F_6
**P**	POF_3
**S**	SO_3
**Cl**	C_2FCl
**Br**	C_2H_3Br
**I**	CH_3I

Table S2. Element-specific reference corrections obtained by aligning DFT-based enthalpies of formation at 298 K to experimentally evaluated thermochemical data using the pMuTT framework. Reported values (in Hartree) represent cumulative molecular reference offsets applied to ensure internal consistency across the dataset and should not be interpreted as isolated atomic energies.

Element	Reference Energy (Ha)
**H**	-6.0971E+02
**C**	-4.0340E+04
**N**	-5.7963E+04
**O**	-7.9554E+04
**F**	-1.0561E+05
**P**	-3.6145E+05
**S**	-4.2164E+05
**Cl**	-4.8741E+05
**Br**	-2.7268E+06
**I**	-3.1456E+05

Table S3. Dataset-wide root-mean-square deviation (RMSD) values for enthalpy, entropy, and temperature-dependent heat capacities obtained from 10-fold cross-validated group-additivity regression using the expanded fluorinated pGrAdd framework. Reported RMSDs quantify aggregate predictive performance across the full fluorocarbon and PFAS dataset prior to application of long-range corrections and do not reflect errors for individual molecules.

Thermodynamic Term	RMSD
**H (kcal·mol^{-1})**	8.1367
**S (cal·mol^{-1}K^{-1})**	10.0524
**Cp (300K) (cal·mol^{-1}K^{-1})**	1.0924
**Cp (400K)**	1.2286
**Cp (500K)**	1.3437
**Cp (600K)**	1.4028
**Cp (700K)**	1.4344
**Cp (800K)**	1.4737
**Cp (900K)**	1.5475
**Cp (1000K)**	1.5880
**Cp (1100K)**	1.6490
**Cp (1200K)**	1.6647
**Cp (1300K)**	1.6925
**Cp (1400K)**	1.6422
**Cp (1500K)**	1.7089

Table S4. Highest maximum absolute error structures (MaxAE) (enthalpy) for pseudo-inverse linear regression of fluorinated structure list. MaxAE was used to check errors in structures and groups to ensure accuracy in group fragmentation and prediction.

Structure	Groups	Occurrence	MaxAE (kcal·mol^{-1})
**FC1(F)C(=O)N(Br)C(=O)C1(F)F**	F(C)	24396	141.90
	C(C)(C[d])(F)2	788	
	C[d](C)(N)(O[d])	59	
	O[d](C[d])	815	
	N(Br)(C[d])2	1	
	Br(N)	1	
**[O-][N+](=O)C(F)(F)C(F)([N+]([O-])=O)[N+]([O-])=O**	O[-](N[+])	19	119.08
	N[+](C)(O[-])(O[d])	12	
	O[d](N[+])	19	
	C(C)(F)2(N[+])	6	
	F(C)	24396	
	C(C)(F)(N[+])2	1	
**FC(=NBr)C(F)(F)C(F)(F)F**	F(C[d])	722	119.07
	C[d](C)(F)(N[d])	8	
	N[d](Br)(C[d])	1	
	Br(N[d])	1	
	C(C)(C[d])(F)2	788	
	F(C)	24396	
	C(C)(F)3	2143	
**FC(F)(F)C(F)(F)P(Cl)Cl**	F(C)	24396	104.94
	C(C)(F)3	2143	
	C(C)(F)2(P)	7	
	P(C)(Cl)2	1	
	Cl(P)	2	
**C(#N)(S(Br))**	C[t](N[t])(S)	5	101.54
	N[t](C[t])	43	
	S(Br)(C[t])	1	
	Br(S)	1	

Table S5. Highest maximum absolute error structures (MaxAE) (enthalpy) for pGrAdd GAF library predictions of fluorinated structure list. MaxAE was used to check errors in structures and groups to ensure accuracy in group fragmentation and prediction.

Structure	Groups	Occurrence	MaxAE (kcal·mol^{-1})
**FC(F)(F)C(F)(F)C(F)(F)C(F)(F)C=CC(C(F)(F)F)(C(F)(F)F)C(F)(F)F**	F(C)	24396	8.83
	C(C)(F)3	2143	
	C(C)2(F)2	5446	
	C(C)(C[d])(F)2	788	
	C[d](C)(C[d])(H)	324	
	C(C)3(C[d])	13	
	Cis	46	
	tbCis	1	
**FC(F)(F)C=CC(F)(F)C(F)(F)C(F)(F)C(F)(F)F**	F(C)	24396	7.89
	C(C[d])(F)3	112	
	C[d](C)(C[d])(H)	324	
	C(C)(C[d])(F)2	788	
	C(C)2(F)2	5446	
	C(C)(F)3	2143	
	Cis	46	
**COC(=O)C=C(O)C(F)(F)C(F)F**	O(C)(C[d])	219	7.71
	C[d](C[d])(O)(O[d])	42	
	O[d](C[d])	815	
	C[d](C[d])2(H)	203	
	C[d](C)(C[d])(O)	24	
	C(C)(C[d])(F)2	788	

	F(C)	24396	
	C(C)(F)2(H)	428	
	C(C)(H)3	940	
	O(C)(H)	580	
	Ether Oxygen Gauche	545	
	Cis	46	
**FC(F)(F)C(F)(F)C=CC(F)(F)C(F)(F)C(F)(F)C(F)(F)C(F)(F)F**	F(C)	24396	7.71
	C(C)(F)3	2143	
	C(C)(C[d])(F)2	788	
	C[d](C)(C[d])(H)	324	
	C(C)2(F)2	5446	
	Cis	46	
**CCN(CC)C(=O)C(F)(F)C(F)F**	C(C)(H)3	940	7.36
	C(C)(H)2(N)	125	
	N(C)2(C[d])	16	
	C[d](C)(N)(O[d])	59	
	O[d](C[d])	815	
	C(C)(C[d])(F)2	788	
	F(C)	24396	
	C(C)(F)2(H)	428	

Table S6. Comparison of like-structures containing varying degrees of group occurrence attributing to the absolute error in prediction accuracy of the regression model. Comparison structure FC1(F)C(=O)NC(=O)C1(F)F was chosen as it was already present in the dataset and differed only by one atom to FC1(F)C(=O)N(Br)C(=O)C1(F)F.

**Structure**	**Groups and Occurrences**	**Actual Enthalpy (kcal·mol^{-1})**	**Predicted Enthalpy (regression) (kcal·mol^{-1})**	**Absolute Error (kcal·mol^{-1})**
FC1(F)C(=O)N(Br)C(=O)C1(F)F 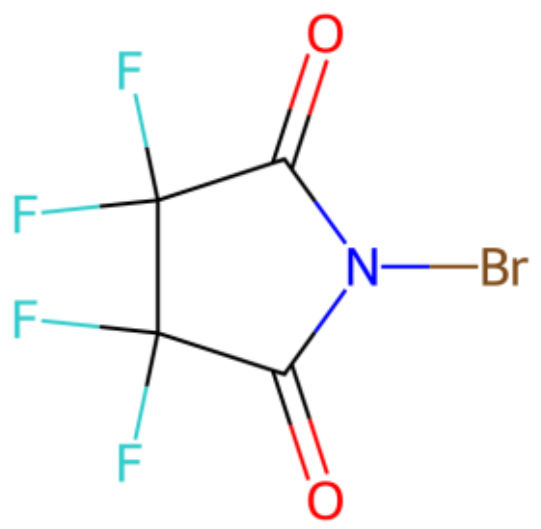	F(C[d]) - 722<br>C[d](C)(F)(N[d]) - 8<br>N[d](Br)(C[d]) - 1<br>Br(N[d]) - 1<br>C(C)(C[d])(F)2 - 788<br>F(C) - 24396<br>C(C)(F)3 - 2143	-163.90	-282.97	119.07
FC1(F)C(=O)NC(=O)C1(F)F 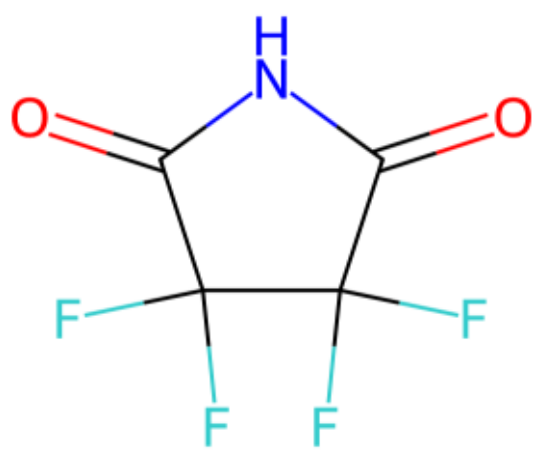	F(C) - 24396<br>C(C)(C[d])(F)2 - 788<br>C[d](C)(N)(O[d]) - 59<br>O[d](C[d]) - 815<br>N(C[d])2(H) - 23	-254.58	-261.26	6.68

Table S7. Experimental gas-phase enthalpies of formation at 298 K for fluorinated hydrocarbons obtained from the NIST Chemistry WebBook and associated literature sources. These species were used for comparison with predictions from the expanded fluorinated pGrAdd framework and to provide direct benchmark comparison to classical Benson-type group additivity schemes.

NIST Reference Species	Enthalpy ($kcal mol^{-1}$)	Reference
**CH_3-CH_2F**	-65.1386	(13)
**CH_2F-CH_2F**	-107.0674	(14)
**CH_3-CHF_2**	-120.2220	(14)
**CH_2F-CHF_2**	-159.7443	(14)
**CH_3-CF_3**	-180.1960	(14)
**CHF_2-CHF_2**	-210.5306	(14)
**CH_2F-CF_3**	-217.4235	(14)
**CHF_2-CF_3**	-266.4460	(14)
**CF_3-CF_3**	-321.5846	(14)
**CH_2=CHF**	-34.1611	(14)
**CH_2=CF_2**	-83.9077	(14)
**CHF=CF_2**	-116.5966	(14)
**CF_2=CF_2**	-161.1616	(13)

Table S8. Comparison of predicted enthalpies of formation from the expanded fluorinated pGrAdd group-additivity framework and Benson group-additivity framework with experimental and high level composite quantum chemical results (B3LYP/def2-TZVP-D3(BJ) vibrational analyses, DF-MP2/aug-cc-pVQZ geometries, and local CCSD(T)/aug-cc-pVQZ single-point energies) reported by Paulechka et. al1. Agreement with both experiment and high-level computation provides and independent benchmark and indicates good transferability within the small-chain fluorinated domain.

Compound	ΔH°_f exp. (298 K) (kcal·mol^{-1})	ΔH°_f calc. (298 K) (kcal·mol^{-1})	ΔH°_f GAF pred.(298 K) (kcal·mol^{-1})	Absolute Error (exp.) (kcal·mol^{-1})	Absolute Error (calc.) (kcal·mol^{-1})
**1,1-Difluoroethane**	-120.46	-120.58	-120.18	0.28	0.40
**1,1,1-Trifluoroethane**	-178.92	-180.16	-175.80	3.12	4.37
**1,1,2-trifluoroethane**	-160.01	—	-161.25	-1.23	—
**Pentafluoroethane**	—	-266.54	-266.46	—	0.08
**Hexafluoroethane**	-321.22	-321.58	-322.08	-0.85	-0.49
**1-Fluoropropane**	-68.07	-70.10	-73.74	-5.67	-3.64
**2-Fluoropropane**	-69.84	-75.02	-77.93	-8.09	-2.90
**2,2-Difluoropropane**	-132.55	—	-125.78	6.78	—
**1,1,1,3,3,3-Hexafluoropropane**	-336.07	-336.81	-325.22	10.84	11.58
**Octafluoropropane**	-426.31	-420.10	-418.33	7.98	1.77
**Vinyl fluoride**	-33.15	-34.32	-30.92	2.23	3.40
**Vinylidene fluoride**	-79.88	-83.91	-79.13	0.75	4.79
**Trifluoroethylene**	-116.28	-119.17	-119.71	-3.43	-0.54
**Tetrafluoroethylene**	-160.85	—	-167.92	-7.07	—
**CF3CH=CH2 (3,3,3-Trifluoropropene)**	-148.59	-150.50	-142.91	5.68	7.60
**CF3CF=CF2 (Hexafluoropropene)**	-275.29	-275.00	-273.06	2.23	1.94

Table S9. Comparison of predicted enthalpies of formation from the expanded fluorinated pGrAdd group-additivity framework with high level composite quantum chemical results (DLPNO-CCSD(T)/CBS) reported in literature by Melin et.al.[12] Data provides a benchmark for the pGrAdd GAF library against a range of functional groups (alkane, alcohol and alkanoic acid) as well as chain length (C2-C8).

Species	Compound	DLPNO-CCSD(T)/CBS (kcal·mol^{-1})	pGrAdd pred. ((kcal·mol^{-1})	Absolute Error (kcal·mol^{-1})
**CF3COOH**	Trifluoroacetic acid	-245.60	-245.93	-0.33
**C2F5COOH**	Pentafluoropropionic acid	-345.20	-345.35	-0.15
**C3F7COOH**	Heptafluorobutyric acid	-441.40	-441.61	-0.21
**C4F9COOH**	Perfluoropentanoic acid	-539.60	-537.87	1.73
**C5F11COOH**	Perfluorohexanoic acid	-637.00	-634.13	2.87
**C6F13COOH**	Perfluoroheptanoic acid	-734.60	-730.39	4.21
**C7F15COOH**	Perfluorooctanoic acid	-832.40	-826.65	5.75
**C2F6**	Hexafluoroethane	-320.70	-322.08	-1.38
**C3F8**	Octafluoropropane	-418.30	-418.33	-0.03
**C4F10**	Decafluorobutane	-515.60	-514.59	1.01
**C5F12**	Dodecafluoropentane	-612.80	-610.85	1.95
**C6F14**	Tetradecafluorohexane	-710.30	-707.11	3.19
**C7F16**	Hexadecafluoroheptane	-807.10	-803.37	3.73
**C8F18**	Octadecafluorooctane	-905.00	-899.63	5.37
**CF3CH2OH**	2,2,2-Trifluoroethanol	-213.60	-213.41	0.19
**C2F5CH2OH**	2,2,3,3,3-Pentafluoropropan-1-ol	-312.90	-309.66	3.24
**C3F7CH2OH**	2,2,3,3,4,4,4-Heptafluorobutan-1-ol	-410.00	-405.92	4.08
**C4F9CH2OH**	Perfluoropentan-1-ol	-506.10	-502.18	3.92
**C5F11CH2OH**	Perfluorohexan-1-ol	-602.70	-598.44	4.26
**C6F13CH2OH**	Perfluoroheptan-1-ol	-699.90	-694.70	5.20
**C7F15CH2OH**	Perfluorooctan-1-ol	-797.20	-790.96	6.24

S8. Supplementary Figures

Supplementary Figures S1–S4 provide illustrative examples of the grammar-based fragmentation rules, diagnostic outlier analysis, and independent computational benchmarks supporting the analyses described in Sections S2 and S3.

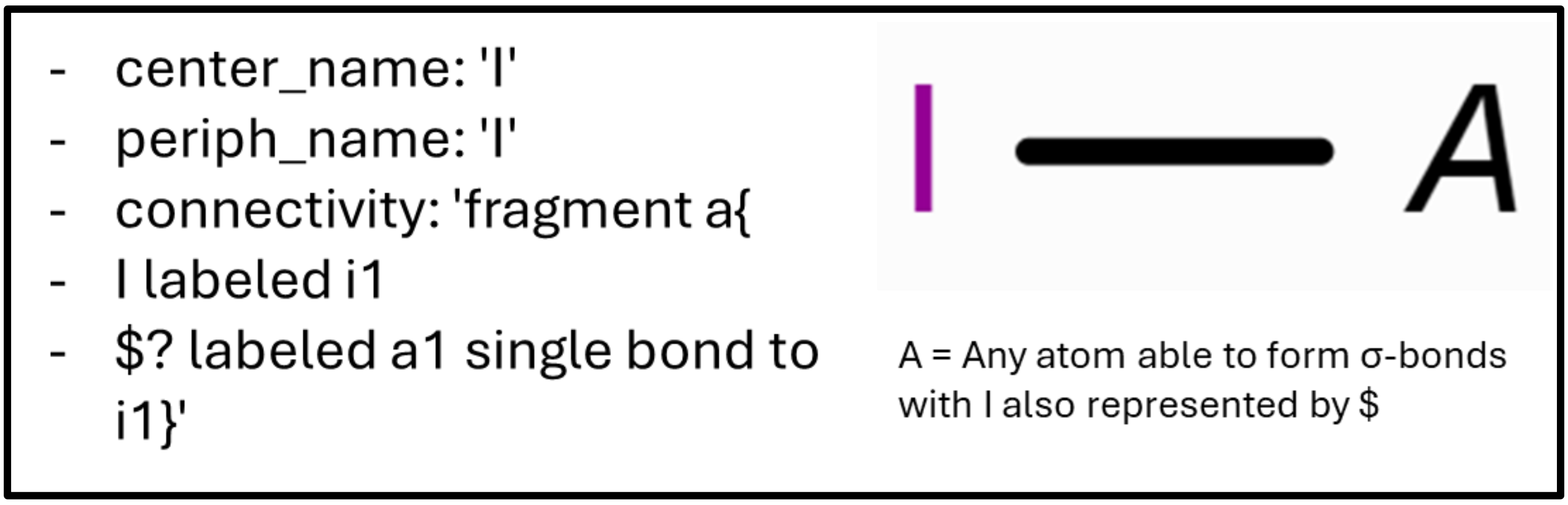

Figure S1. Example of Silver language rulesets used to identify fragments for an iodine center illustrating the definition of halogenated local environments within the fluorinated group-additivity framework. Groups are defined using an extensible attribute-grammar formalism that systematically captures fluorinated substitution patterns while preventing overlap or misrepresentation of chemically distinct environments.

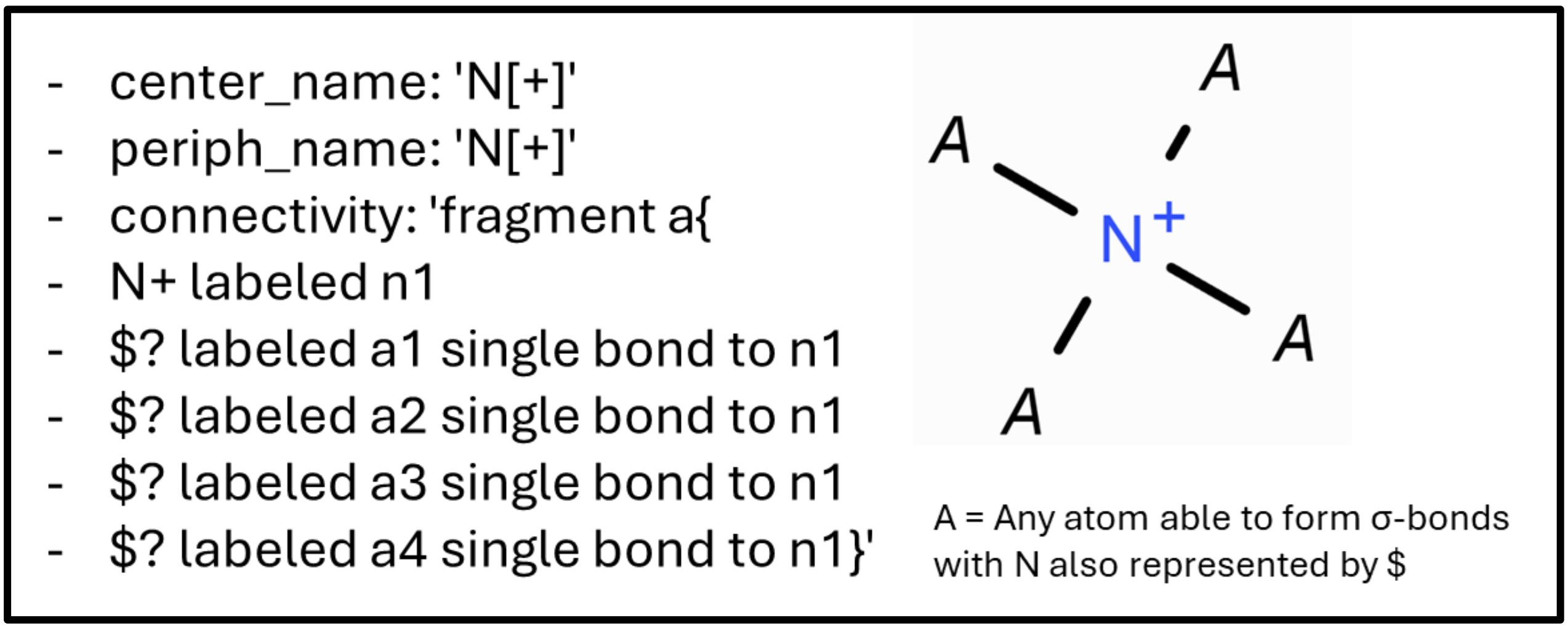

Figure S2. Example of Silver language rulesets used to identify fragments for cationic nitrogen environments relevant to PFAS chemistry. The attribute-grammar formalism enables explicit differentiation of bonding patterns, coordination states, and charge environments required for chemically consistent fragmentation of ionized species.

Outlier Identification Using Grubbs' Test

Mean absolute error: μ = 1.9832 kcal/mol
Standard deviation: σ = 1.6134 kcal/mol

Removed point:
Absolute error: 11.7863 kcal/mol
Z-score: 6.08 σ
Grubbs statistic: G = 6.0760
Critical value: G_crit = 4.3039

Conclusion: G > G_crit = The point is a statistically significant outlier.

Molecular entry:
SMILES: OC(=O)C1CCN(CC(F)(F)C(F)F)CC1
DFT enthalpy: -292.3963 kcal/mol
pGrAdd enthalpy: -280.6100 kcal/mol
Absolute error: 11.7863 kcal/mol

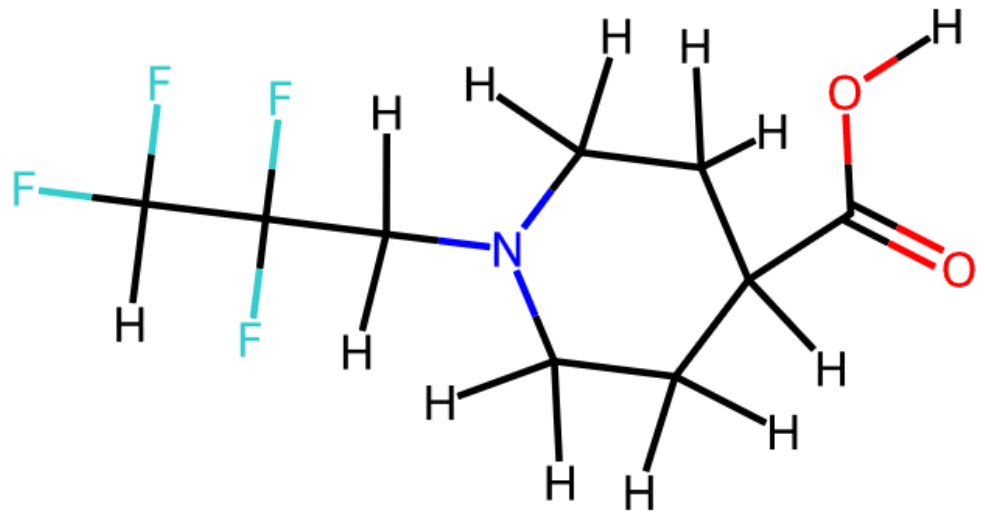

Group:	Total Occurrence:
C[d](C)(O)(O[d]): 1	288
O[d](C[d]): 1	815
C(C)2(C[d])(H): 1	40
C(C)2(H)2: 2	1647
C(C)(H)2(N): 3	125
N(C)3: 1	20
C(C)2(F)2: 1	5446
F(C): 4	24396
C(C)(F)2(H)': 1	428
'O(C)(H)': 1	580
'AlkaneGauche': 2	409

Figure S3. Grubbs' test analysis applied to cross-validated residuals following application of pGrAdd long-range corrections. The test was used as a diagnostic tool to identify statistically anomalous predictions rather than as a definitive exclusion criterion, acknowledging assumptions of approximate normality and single-outlier behavior.

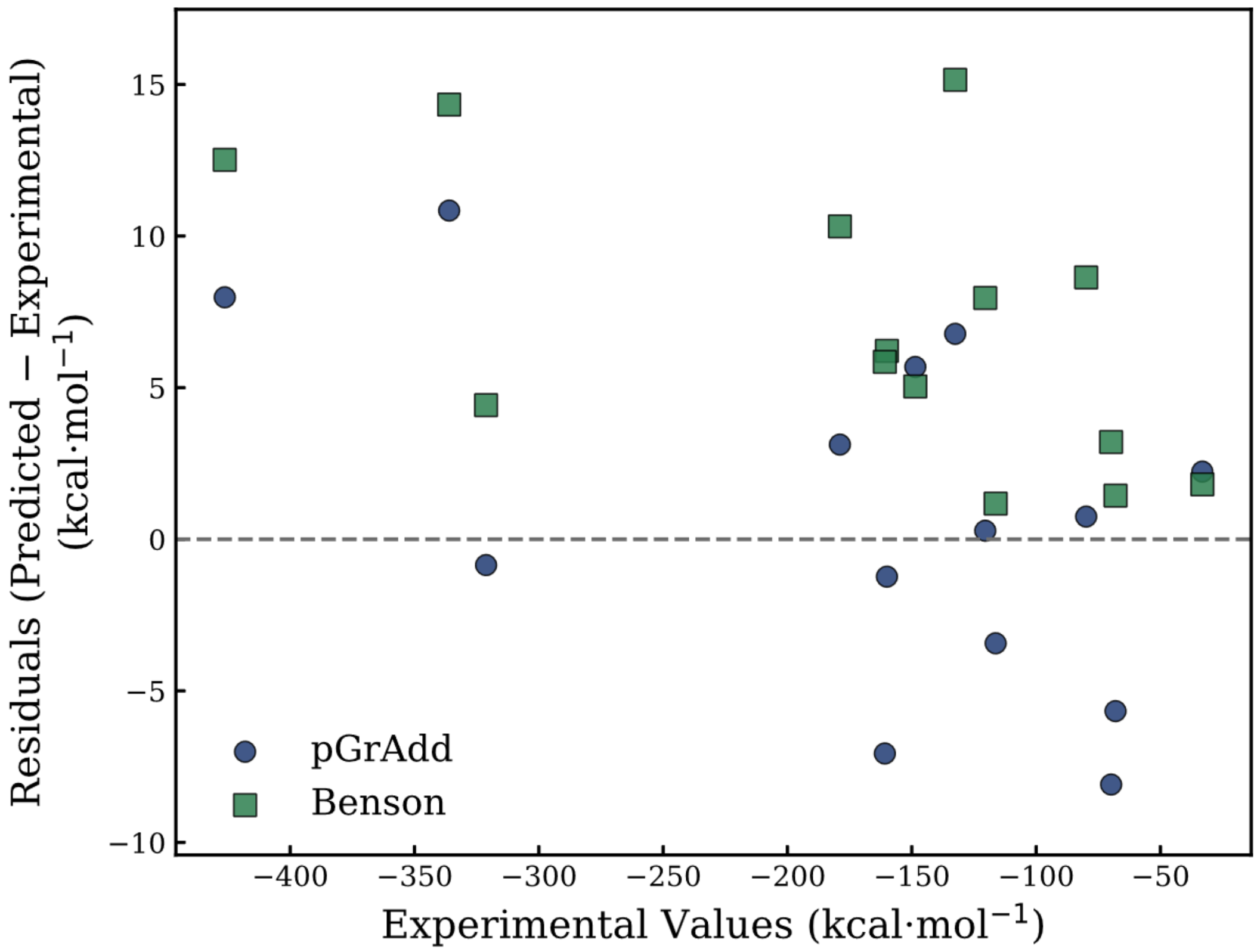

Figure S4. Residual comparison between predicted gas-phase enthalpies of formation and experimental reference values reported by Paulechka et al.[11] Residuals (Predicted − Experimental) are shown for both the expanded fluorinated pGrAdd framework and the original Benson group-additivity scheme. The dashed horizontal line denotes perfect agreement with experiment. Across this experimental dataset, the pGrAdd model exhibits reduced systematic deviation relative to the Benson scheme, yielding an RMSD of 5.58 kcal·mol^{-1} compared to 8.33 kcal·mol^{-1} for the legacy groups. These results demonstrate that the expanded fluorinated group library provides more accurate and consistent thermochemical predictions for fluorinated species.

References

(1) Lym, J.; Wittreich, G. R.; Vlachos, D. G. A Python Multiscale Thermochemistry Toolbox (pMuTT) for Thermochemical and Kinetic Parameter Estimation. *Comput. Phys. Commun.* **2020**, *247*, 106864. https://doi.org/10.1016/j.cpc.2019.106864.

(2) Mcbride, B. J.; Gordon, S.; Reno, M. A. *Coefficients for Calculating Thermodynamic and Transport Properties of Individual Species*; NASA-TM-4513; 1993. https://ntrs.nasa.gov/citations/19940013151 (accessed 2026-04-13).

(3) Shomate, C. H. High-Temperature Heat Contents of Magnesium Nitrate, Calcium Nitrate and Barium Nitrate[1]. *J. Am. Chem. Soc.* **1944**, *66* (6), 928–929. https://doi.org/10.1021/ja01234a025.

(4) Linstrom, P. NIST Chemistry WebBook, NIST Standard Reference Database 69, 1997. https://doi.org/10.18434/T4D303.

(5) Grubbs, F. E. Procedures for Detecting Outlying Observations in Samples. *Technometrics* **1969**, *11* (1), 1–21. https://doi.org/10.1080/00401706.1969.10490657.

(6) Wittreich, G. R.; Vlachos, D. G. Python Group Additivity (pGrAdd) Software for Estimating Species Thermochemical Properties. *Comput. Phys. Commun.* **2022**, *273*, 108277. https://doi.org/10.1016/j.cpc.2021.108277.

(7) Van Wyk, E.; Bodin, D.; Gao, J.; Krishnan, L. Silver: An Extensible Attribute Grammar System. *Sci. Comput. Program.* **2010**, *75* (1), 39–54. https://doi.org/10.1016/j.scico.2009.07.004.

(8) Penrose, R. A Generalized Inverse for Matrices. *Math. Proc. Camb. Philos. Soc.* **1955**, *51* (3), 406–413. https://doi.org/10.1017/S0305004100030401.

(9) Burman, P. A Comparative Study of Ordinary Cross-Validation, *v* -Fold Cross-Validation and the Repeated Learning-Testing Methods. *Biometrika* **1989**, *76* (3), 503–514. https://doi.org/10.1093/biomet/76.3.503.

(10) Stone, M. Cross-Validatory Choice and Assessment of Statistical Predictions. *J. R. Stat. Soc. Ser. B Methodol.* **1974**, *36* (2), 111–133. https://doi.org/10.1111/j.2517-6161.1974.tb00994.x.

(11) Paulechka, E.; Kazakov, A. Critical Evaluation of the Enthalpies of Formation for Fluorinated Compounds Using Experimental Data and High-Level Ab Initio Calculations. *J. Chem. Eng. Data* **2019**, *64* (11), 10.1021/acs.jced.9b00386. https://doi.org/10.1021/acs.jced.9b00386.

(12) Melin, T. R. L.; Harell, P.; Ali, B.; Loganathan, N.; Wilson, A. K. Thermochemistry of Per- and Polyfluoroalkyl Substances. *J. Comput. Chem.* **2023**, *44* (4), 570–580. https://doi.org/10.1002/jcc.27023.

(13) Goos, E.; Burcat, A.; Ruscic, B. (2012) Extended Third Millennium Thermodynamic Database for Combustion and Air-Pollution Use with Updates from Active Thermochemical Tables, Technion-IIT, Haifa, Israel. Available at: Ftp://Ftp.Technion.Ac.Il/Pub/Supported/Aetdd/Thermodynamics/BURCAT.THR. https://respecth.elte.hu/burcat.php (accessed 2026-04-12).

(14) Burgess, D. R.; Zachariah, M. R.; Tsang, W.; Westmoreland, P. R. Thermochemical and Chemical Kinetic Data for Fluorinated Hydrocarbons. *Prog. Energy Combust. Sci.* **1995**, *21* (6), 453–529. https://doi.org/10.1016/0360-1285(95)00009-7.
